# Association Between High-Deductible Health Plans and Disparities in Access to Care Among Cancer Survivors

**DOI:** 10.1001/jamanetworkopen.2020.8965

**Published:** 2020-06-24

**Authors:** Megan B. Cole, Jacqueline E. Ellison, Amal N. Trivedi

**Affiliations:** 1 Department of Health Law, Policy, and Management, Boston University School of Public Health, Boston, Massachusetts; 2 Department of Health Services, Policy, and Practice, Brown University School of Public Health, Boston, Massachusetts

## Abstract

This cross-sectional study uses data from the 2013-2018 National Health Interview Survey to examine the association between enrollment in a high-deductible health plan and access to care among cancer survivors differentiated by race/ethnicity.

## Introduction

Enrollment in high-deductible health plans (HDHPs), which increased from 8% in 2009 to 30% in 2019 among people with employer-sponsored coverage,^1^ is associated with less use of health care, including cancer screening and treatment.^2,3^ High-deductible health plans may have greater consequences for racial/ethnic minorities, given evidence that other forms of cost sharing contribute to disparities in care.^4^ However, few studies have examined the association between HDHPs and racial/ethnic disparities. This study examined the differential association between HDHP enrollment and access to care by race/ethnicity among cancer survivors, a population that often faces substantial cost-related barriers to care.^5^

## Methods

We identified privately insured adults aged 18 to 64 years with a past or current cancer diagnosis (N = 3713, representing 1.16 million patients nationally) using pooled 2013-2018 National Health Interview Survey data. Boston University’s Institutional Review Board deemed the study exempt because the data were publicly available. This study followed the Strengthening the Reporting of Observational Studies in Epidemiology (STROBE) reporting guideline.

Doubly robust ordinary least squares regression models with stabilized inverse probability of treatment weights (IPTWs), which were based on propensity scores and multiplied by survey weights, estimated the association between HDHP enrollment and 8 measures of cost-related barriers to accessing care (eg, skipping medication to save money, inability to afford a specialist). Propensity scores, which balanced on observable covariates for patients with vs without an HDHP, included 16 patient-level sociodemographic and clinical covariates (Table 1). The parameter of interest was an interaction between HDHP enrollment and self-reported race/ethnicity; black vs non-Hispanic white was the main comparison of interest. Models were further adjusted for year and for the covariates included in the propensity score and used robust variance estimators. *P* values were 2-tailed, and statistical significance was set at α = .05. Analyses were performed using Stata statistical software, version 15.0 (StataCorp).

**Table 1.** Unadjusted Characteristics of Cancer Survivors by High-Deductible Health Plan (HDHP) Enrollment Status (2013-2018)^a^

Characteristic	HDHP, No. (%)^b^	
	No (n = 2067)	Yes (n = 1646)
Age, mean (SD), y	54.1 (9.1)	53.5 (9.3)
Race/ethnicity		
Non-Hispanic white	1783 (86.3)	1481 (90.0)
Hispanic	114 (5.5)	71 (4.3)
Non-Hispanic black	117 (5.7)	57 (3.5)
Other	53 (2.6)	37 (2.2)
Sex		
Men	753 (36.4)	553 (33.6)
Women	1314 (63.6)	1093 (66.4)
US census region		
Northeast	409 (19.8)	247 (15.0)
Midwest	442 (21.4)	475 (28.9)
South	685 (33.1)	543 (33.0)
West	531 (25.7)	381 (23.1)
Income as % of federal poverty level		
<200%	230 (11.1)	155 (9.4)
200%-399%	468 (22.6)	400 (24.3)
≥400%	1138 (55.1)	899 (54.6)
Unknown	231 (11.2)	192 (11.7)
Highest level of education		
High school or less	495 (23.9)	345 (21.0)
Associate’s degree or some college	667 (32.3)	574 (34.9)
Bachelor’s degree	536 (25.9)	439 (26.7)
Graduate degree	369 (17.9)	288 (17.5)
Employment status		
Employed	1395 (67.5)	1151 (69.9)
Unemployed	411 (19.9)	337 (20.5)
Retired	261 (12.6)	158 (9.6)
Other characteristics		
English is primary language	2043 (98.8)	1642 (99.8)
Born in the US	1934 (93.6)	1561 (94.8)
Married	1292 (62.5)	986 (59.9)
Self-rated health is “good” or higher	1707 (82.6)	1373 (83.4)
Health conditions (past or current)		
Hypertension	900 (43.5)	638 (38.8)
Diabetes	321 (15.5)	195 (11.8)
Asthma	330 (16.0)	282 (17.1)
Any heart disease	291 (14.1)	259 (15.7)
Liver disease	59 (2.9)	58 (3.5)
Cancer	2067 (100.0)	1646 (100.0)

^a^ Data reflect unadjusted distributions of population characteristics before applying inverse probability of treatment weights (IPTWs). After applying IPTWs, all standardized differences between characteristics of those with vs without an HDHP were less than 0.01.

^b^ As of 2018, an HDHP was defined as having a deductible of at least $1350 for an individual and at least $2700 for a family. Sample sizes reflect survey samples, which represent 522 619 cancer survivors with an HDHP and 636 834 cancer survivors without an HDHP across the US based on pooled 2013-2018 National Health Interview Survey data. Forty-four percent of privately insured cancer survivors were enrolled in an HDHP during the study period (2013-2018), although this percentage ranged from 37% in 2013 to 50% in 2018.

## Results

Among the 3713 cancer survivors whose data were included in the analysis, 2407 (64.8%) were women, 1305 (35.2%) were men, 3264 (87.9%) were non-Hispanic white individuals, and the mean (SD) age was 53.8 (9.2) years. Compared with cancer survivors not enrolled in an HDHP, cancer survivors enrolled in an HDHP were more likely to be non-Hispanic white individuals (1481 of 1646 [90.0%] vs 1783 of 2067 [86.3%]) and less likely to have diabetes (195 of 1646 [11.8%] vs 321 of 2067 [15.5%]) or hypertension (638 of 1646 [38.8%] vs 900 of 2067 [43.5%]) (Table 1); other characteristics were statistically similar. After applying IPTWs, there were no statistical differences in observed characteristics between the 2 groups.

Forty-four percent of privately insured cancer survivors were enrolled in an HDHP during the study period (2013-2018), although this percentage ranged from 37% in 2013 to 50% in 2018. Enrollment in an HDHP was associated with greater cost-related barriers to accessing care when comparing cancer survivors with vs without an HDHP, and the magnitude of the association was statistically significantly larger for black vs white patients for 4 of 8 measures examined (Table 2). For example, 13 of 57 (22.8%) black vs 118 of 1481 (8.0%) white cancer survivors with an HDHP skipped medication to save money, compared with 9 of 117 (7.7%) black vs 97 of 1783 (5.4%) white cancer survivors without an HDHP (adjusted interaction coefficient = 17.6 percentage points; 95% CI, 3.8-31.3; *P* = .01). Enrollment in an HDHP also had a greater adverse association with black vs white patients when assessing the use of less medication to save money (adjusted interaction coefficient = 19.7 percentage points; 95% CI, 5.8-33.6; *P* = .006), delays in filling a prescription to save money (adjusted interaction coefficient = 15.4 percentage points; 95% CI, 0.1-30.7; *P* = .049), and being unable to afford a specialist (adjusted interaction coefficient = 16.9 percentage points; 95% CI, 1.2-32.7; *P* = .04).

**Table 2.** Differential Associations Between HDHP Enrollment and Cost-Related Barriers to Care by Race/Ethnicity Among Cancer Survivors (2013-2018)

Outcome	HDHP, unadjusted rates (%)^a^		Adjusted results with IPTWs, coefficient (95% CI)^b^	*P* value
	No (n = 2067)	Yes (n = 1646)		
Skipped medication to save money				
White	97/1783 (5.4)	118/1481 (8.0)	1 [Reference]	
Hispanic	7/114 (6.1)	5/71 (7.0)	0.9 (−7.6 to 9.3)	.84
Black	9/117 (7.7)	13/57 (22.8)	17.6 (3.8 to 31.3)	.01
Other	2/53 (3.8)	6/37 (16.2)	5.7 (−6.3 to 17.67)	.35
Took less medicine to save money				
White	97/1783 (5.4)	128/1481 (8.6)	1 [Reference]	
Hispanic	7/114 (6.1)	8/71 (11.3)	4.2 (−5.4 to 13.8)	.39
Black	10/117 (8.5)	14/57 (24.6)	19.7 (5.8 to 33.6)	.006
Other	2/53 (3.8)	5/37 (13.5)	4.9 (−7.2 to 16.9)	.43
Delayed filling prescription to save money				
White	126/1783 (7.1)	114/1481 (7.7)	1 [Reference]	
Hispanic	10/114 (8.8)	10/71 (14.1)	1.4 (−10.5 to 13.2)	.82
Black	19/117 (16.2)	16/57 (28.1)	15.4 (0.1 to 30.7)	.049
Other	2/53 (3.8)	7/37 (18.9)	7.6 (−6.3 to 21.4)	.28
Used alternative therapies to save money				
White	78/1783 (4.4)	114/1481 (7.7)	1 [Reference]	
Hispanic	9/114 (7.9)	8/71 (11.3)	2.4 (−9.5 to 14.3)	.69
Black	5/117 (4.3)	9/57 (15.8)	12.1 (−2.1 to 26.2)	.09
Other	3/53 (5.7)	4/37 (10.8)	2.5 (−10.7 to 15.8)	.71
Medical care delayed owing to cost				
White	159/1783 (8.9)	214/1481 (14.4)	1 [Reference]	
Hispanic	15/114 (13.2)	14/71 (19.7)	−5.7 (−19.7 to 8.2)	.42
Black	18/117 (15.4)	13/57 (22.8)	7.1 (−7.7 to 21.9)	.35
Other	5/53 (9.4)	5/37 (13.5)	−9.8 (−25.5 to 5.9)	.22
Needed and did not get care owing to cost				
White	92/1783 (5.2)	119/1480 (8.0)	1 [Reference]	
Hispanic	8/114 (7.0)	9/71 (12.7)	1.6 (−11.3 to 14.5)	.81
Black	11/117 (9.4)	9/57 (15.8)	3.4 (−9.5 to 16.3)	.61
Other	2/53 (3.8)	4/37 (10.8)	−2.4 (−15.3 to 10.4)	.71
Could not afford specialist^c^				
White	45/1553 (2.9)	76/1234 (6.2)	1 [Reference]	
Hispanic	7/99 (7.1)	6/62 (9.7)	3.4 (−8.7 to 15.4)	.58
Black	5/102 (4.9)	7/47 (14.9)	16.9 (1.2 to 32.7)	.04
Other	1/46 (2.2)	2/31 (6.5)	−0.8 (−10.4 to 8.8)	.87
Could not afford follow-up care^c^				
White	40/1553 (2.6)	60/1234 (4.9)	1 [Reference]	
Hispanic	6/99 (6.1)	6/62 (9.7)	1.1 (−12.4 to 14.6)	.87
Black	5/102 (4.9)	5/47 (10.6)	12.5 (−2.9 to 27.9)	.11
Other	1/46 (2.2)	0/31 (0.0)	−5.7 (−11.9 to 0.5)	.07

Abbreviations: HDHP, high-deductible health plan; IPTWs, inverse probability of treatment weights.

^a^ As of 2018, an HDHP was defined as having a deductible of at least $1350 for an individual and at least $2700 for a family. Sample sizes reflect survey samples, which represent 522 619 cancer survivors with an HDHP and 636 834 cancer survivors without an HDHP across the US based on pooled 2013-2018 National Health Interview Survey data. Forty-four percent of privately insured cancer survivors were enrolled in an HDHP during the study period (2013-2018), although this percentage ranged from 37% in 2013 to 50% in 2018.

^b^ Coefficients represent the coefficient of the interaction term between an indicator for HDHP enrollment and race/ethnicity based on a fully adjusted linear regression model that uses IPTWs; IPTWs are calculated based on propensity scores generated from a logistic regression model. All coefficients are reported as absolute percentage point differences relative to white non-Hispanic patients (reference group). A coefficient greater than 0 implies that the access-related racial/ethnic disparity among patients with an HDHP was greater than the disparity among patients without an HDHP.

^c^ Questions were not reported in the 2018 National Health Interview Survey; thus, sample sizes and denominators are smaller.

## Discussion

We found that HDHPs were associated with cost-related barriers to care for cancer survivors, and these barriers were significantly greater for black patients. These findings align with those of studies showing that HDHPs reduce access to and use of cancer-related services^2,3^ and add to the only known prior study on racial/ethnic disparities in HDHPs, which found no racial/ethnic differences in cardiovascular medication adherence owing to HDHP enrollment.^6^ Our study limitations included potential unobserved confounding between those with vs without an HDHP, small samples for racial subgroups, and lack of information on plan options. Nonetheless, this study found that the magnitude of association between HDHPs and cost-related barriers to care is larger among black cancer survivors relative to their white counterparts, elucidating the potential for HDHPs to widen documented racial/ethnic disparities in cancer outcomes.
